# Correction to: Effects of different preservation methods of human iliac veins

**DOI:** 10.1007/s10561-022-10058-w

**Published:** 2022-12-10

**Authors:** Zhang-Yong Ren, Bing Pan, Fang-Fei Wang, Shao-Cheng Lyu, Qiang He

**Affiliations:** Department of Hepatobiliary Surgery, Beijing Chao-Yang Hospital, Capital Medical University, Beijing, 100020 China

**Correction to: Cell Tissue Bank**

10.1007/s10561-022-10055-z

The article “Effects of different preservation methods of human iliac veins”, written by Zhang-Yong Ren, Bing Pan, Fang-Fei Wang, Shao-Cheng Lyu, Qiang He, was originally published online on the publisher’s internet portal on 28 November 2022 with Open Access under a Creative Commons Attribution 4.0 International License.

With the author’s/authors' decision to cancel Open Access the copyright of the article changed on 29 November 2022 to © The Author(s), under exclusive licence to Springer Nature B.V. 2022 with all rights reserved.

